# A cross-sectional study on the association between dietary inflammatory index and hyperuricemia based on NHANES 2005–2018

**DOI:** 10.3389/fnut.2023.1218166

**Published:** 2023-09-21

**Authors:** Hao Wang, Shengmei Qin, Feng Li, Huanhuan Zhang, Ling Zeng

**Affiliations:** ^1^Department of Critical Care Medicine, West China Hospital, Sichuan University, Chengdu, China; ^2^West China School of Nursing, Sichuan University, Chengdu, China; ^3^Department of Nursing, Stomatological Hospital Southern Medical University, Guangzhou, China

**Keywords:** dietary inflammatory index, hyperuricemia, adults, the United States, NHANES

## Abstract

**Background:**

Hyperuricemia is a common condition that can lead to gout and other related diseases. It has been suggested that Inflammatory factors play important role in the development and progression of hyperuricemia. The dietary inflammatory index (DII) enables the assessment of the inflammatory potential of an individual’s diet. This study aimed to investigate the association between DII and hyperuricemia.

**Methods:**

This study was performed based on a cross-sectional dataset from the National Health and Nutrition Examination Survey (NHANES) 2005–2018. Participants aged 18 years and above with dietary intake and serum uric acid level information were included. DII scores were calculated using dietary intake data, based on which participants were categorized into tertiles. Multivariable logistic regression analysis was adopted to investigate the association between DII and hyperuricemia.

**Results:**

Among a total of 31,781 participants in the analysis, 5,491 had hyperuricemia. After adjusting confounding factors, the odds of hyperuricemia are significantly higher in the second (OR 1.17, 95% CI 1.07–1.29) and third tertiles (OR 1.31, 95% CI 1.19–1.44) relative to the first one.

**Conclusion:**

This study suggested that diet with higher inflammatory potential, as measured by DII, is associated with increased hyperuricemia risk. These findings indicated that dietary modification may be a potential approach for hyperuricemia’s prevention and control.

## 1. Introduction

Hyperuricemia is defined as the overproduction or under-excretion of uric acid, and it tops the list of global disease burdens associated with gout and a wide spectrum of other diseases, affecting patients of all ages and sexes (1, 2). It is an independent risk factor for various systemic diseases, including gout, cardiovascular diseases, hypertension, chronic kidney disease, and many others (3). Up to 2016, the global prevalence of hyperuricemia has hit 21% (4). Its prevalence in U.S. ranged from 14.6 to 20% (5) and showed a tendency to affect younger people (38.6 ± 11.8 years), even non-obese adults (6, 7).

Unhealthy eating habit is closely related to hyperuricemia since its influence on inflammation (8). For instance, Western dietary patterns characterized by high caloric content and significant fat levels have been linked to an increased presence of inflammatory markers in the body (9). Correspondingly, anti-inflammatory diets could reduce this level, and dietary management targeting at asymptomatic hyperuricemia may be of great benefit (10).

Since inflammation is an essential factor for hyperuricemia, measuring inflammation level might be useful for the prediction of and protection against hyperuricemia. The Dietary Inflammatory Index (DII) was firstly proposed by Shivappa et al. in 2009 based on published literatures (11) and was updated in 2014 (12). It specifically aimed to measure dietary inflammation potential. DII, as is closely related to the expression of blood inflammatory markers, has been widely used in the investigation of the association between inflammation caused by diet and the onset and progression of diverse ailments (13).

Up to now, there are only two studies on the association between DII and hyperuricemia. One was a cross-sectional study in China, whose results showed that higher DII scores were associated with higher hyperuricemia risk after covariates adjustment (14). The other was a case–control study in Korea, which reported that higher pro-inflammatory dietary intake was significantly associated with the risk of hyperuricemia only in males (6). However, both studies focused on Asian population, and can only provide limited reference for U.S. population due to the differences between Eastern and Western dietary habits (15). This study aimed to explore such a relationship in U.S. population.

## 2. Materials and methods

### 2.1. Study population

**Figure 1:**
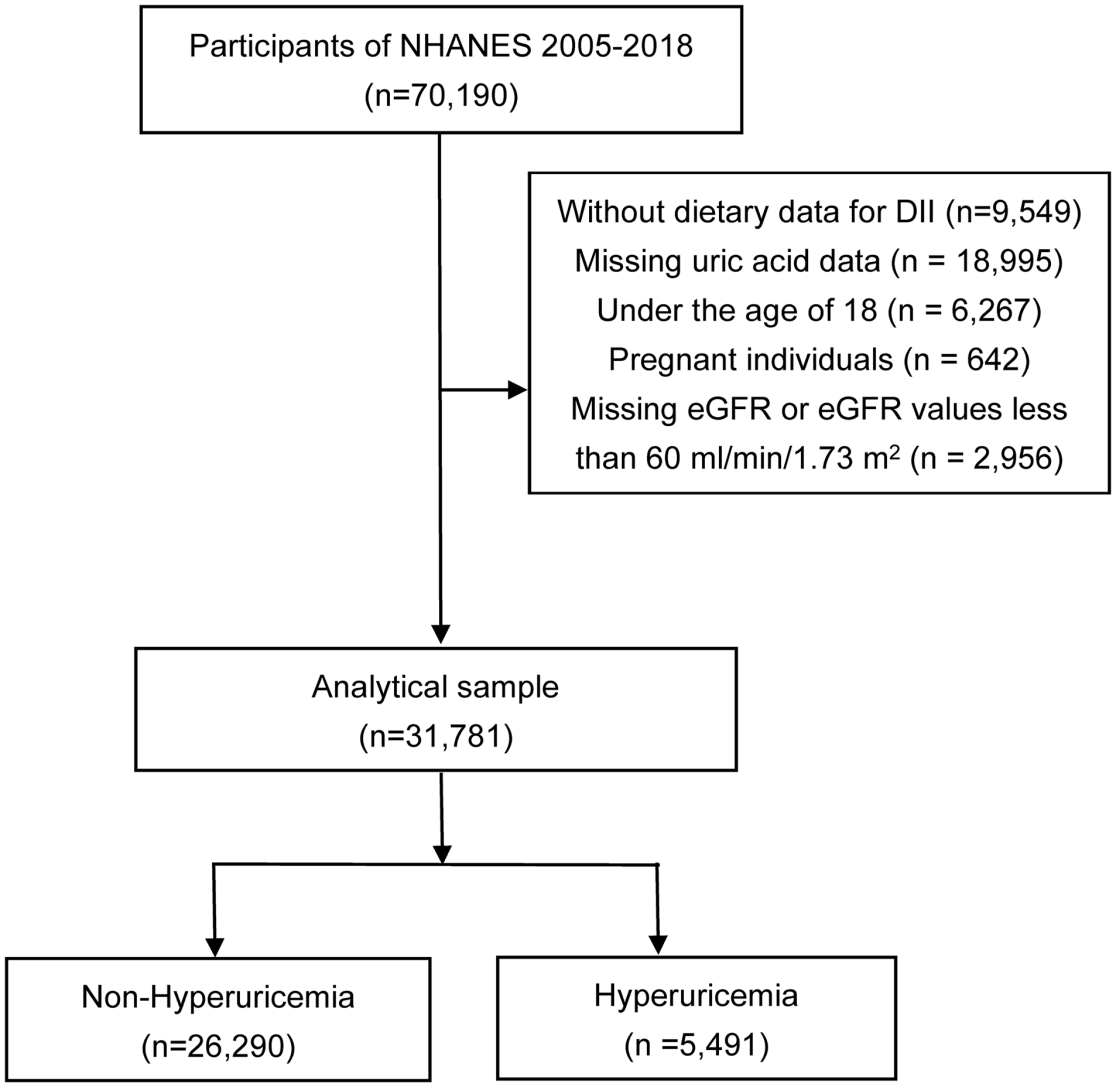
Flowchart of the study. NHANES, the National Health and Nutrition Examination Survey; DII, dietary inflammatory index; eGFR, estimated glomerular filtration rate.

The National Health and Nutrition Examination Survey (NHANES) is a sweeping cross-sectional study administered by the National Center for Health Statistics. Its objective is to compile crucial data about individuals’ health conditions by conducting a range of interviews, physical assessments, and laboratory examinations. To ensure an accurate representation of the overall U.S. population, a sophisticated multistage sampling technique was employed for this particular survey. In order to evaluate the dietary patterns of NHANES participants, two comprehensive interviews were conducted to obtain dietary-recall information. Specifically, the first interview was implemented face-to-face by highly trained dietary interviewers in NHANES Mobile Examination Center (MEC), while the second interview was conducted over phone three to ten days after the MEC interview (the next week of MEC interview). During the dietary-recall interviews, U.S. Department of Agriculture Automated Multiple-Pass Method dietary interview approach was used to collect information on food intake. Participants utilized measuring guides during in-person interviews while a food model booklet was used during the telephone interviews to quantify food. The methodology and materials used for the survey underwent ethical review and were approved by the Ethics Review Board of the National Center for Health Statistics. Further, prior to participating in the survey, written informed consent was obtained from all individuals involved. Data from NHANES 2005–2018 cycle were selected in this study, including a total of 70,190 participants initially. Exclusion criteria were as follows: (a) participants without dietary data for DII calculation (*n* = 9,549), (b) participants with missing uric acid data (*n* = 18,995), (c) individuals under the age of 18 years old (*n* = 6,267), (d) pregnant individuals (*n* = 642), and (e) individuals with missing estimated glomerular filtration rate (eGFR) or eGFR values less than 60 mL/min/1.73 m (2) (*n* = 2,956). Ultimately, 31,781 participants were included in the analysis, as depicted in Figure 1.

### 2.2. Calculation of DII

DII developed by Shivappa et al. was computed to measure the inflammatory potential of different dietary patterns (12). DII calculation was conducted by a standardized global database that contains daily dietary intake information of 11 regionally representative populations. Both the standard mean and standard deviation were provided for all DII food parameters from the world database. To ensure that the investigation is thorough and accurate, a scoring system was created. A score of “+1” is given to dietary components that increase the levels of CRP, TNF-a, IL-1b, and IL-6, or decrease the levels of IL-4 and IL-10. Conversely, a score of “−1” is assigned to dietary components that reduce the levels of CRP, TNF-a, IL-1b, and IL-6, or increase the levels of IL-4 and IL-10. This comprehensive approach allows for a more detailed examination of how dietary components affect these specific indicators. These values were weighted according to the study design. The z-score for each food parameter was calculated by subtracting the standard mean from the value of consumption reported by each individual and then dividing that result by the standard deviation. These z-scores were transformed into proportions (ranging from 0 to 1) to minimize the effect of positive skewing. To obtain a symmetrical distribution centered around zero with bounds between −1 and + 1, each proportion was doubled, and then 1 was subtracted. This value was then multiplied by the corresponding inflammatory effect score for each food parameter. In this study, there were 28 parameters available in NHANES data that could be utilized to calculate DII, including energy, protein, carbohydrate, dietary fiber, total fatty acid, total saturated fatty acid, monounsaturated fatty acids (MUFA), polyunsaturated fatty acids (PUFA), cholesterol, β-carotene, niacin, folate, magnesium, iron, zinc, selenium, caffeine, alcohol, n3 polyunsaturated fatty acid, n6 polyunsaturated fatty acid, and vitamins A, B1, B2, B6, B12, C, D, and E. An elevated DII score signifies the consumption of a diet that triggers inflammation, whereas a reduced score indicates the adoption of an anti-inflammatory diet.

### 2.3. Serum uric acid measurement

This investigation was primarily focused on hyperuricemia condition, which is characterized by elevated uric acid level in bloodstream. The measurement of serum uric acid level was carried out using the Beckman UniCel® DxC800 Synchron or Beckman Synchron LX20 (Beckman Coulter, Inc., Brea, CA, United States), which employs an oxidizing process to convert uric acid to allantoin and H2O2. Following established diagnostic standards, hyperuricemia was delineated as a serum uric acid threshold of 7.0 mg/dL or more in males and 6.0 mg/dL or more in females (16).

### 2.4. Covariates

In this study, demographic, lifestyle variables, physical measurements, laboratory tests, and self-reported health status were assessed in a computer-assisted personal interview. Demographic information includes age, sex, race/ethnicity, and educational level; health status contains smoking, drinking, physical activity, and disease history (hypertension, diabetes and hyperlipidemia); physical health examination involves height, body mass, and blood pressure measurements; and laboratory tests covers uric acid, serum glucose level.

The smoking status was divided into three distinct groups for this study. The first group consisted of individuals who had never smoked, meaning that they had smoked less than 100 cigarettes in their entire lifetime. The second group comprised individuals who were former smokers, having smoked more than 100 cigarettes in their lifetime and subsequently quit smoking at the time of the survey. Finally, the third group consisted of current smokers, who had smoked more than 100 cigarettes in their lifetime and continued to smoke at least every few days. Current drinking was classified into three categories: heavy drinking (≥3 drinks per day for females; ≥4 drinks per day for males; binge drinking on 5 or more days per month), moderate drinking (≥2 drinks per day for females; ≥3 drinks per day for males; binge drinking ≥2 days per month), and mild drinking (others). To determine the individual’s metabolic equivalent of task (MET)/week, a calculation was performed by multiplying the total number of minutes spent on various activities during the week by the metabolic equivalents estimated by the Compendium of Physical Activities. This approach allowed for an accurate assessment of the intensity and frequency of the individual’s physical activity over the course of the week. By utilizing the Compendium of Physical Activities, which provides standardized estimates of metabolic equivalents for various activities, the calculation was based on scientifically informed data and avoided potential inaccuracies and bias. The level of physical activity was determined in terms of hours of activity per week (MET/week), and results were divided into three groups: low (<600 METs/week), moderate (600–1,199 METs/week), and vigorous (≥1,200 METs/week). The eGFR was determined through the application of the Chronic Kidney Disease Epidemiology Collaboration creatinine equation. This formula calculates eGFR values based on specific parameters for both male and female. For male, the equation is as follows: eGFR = (140-age) × body weight (kg) × 1.23/creatinine (mmol/L). Similarly, for female, the equation is: eGFR = (140-age) × body weight (kg) × 1.03/creatinine (mmol/L). Hypertension was defined as blood pressure ≥ 140/90 mmHg, a diagnosis of hypertension, or a prescription of antihypertensive drugs in health questionnaire. Diabetes was diagnosed when patients met one or more of the following criteria: (1) a medical diagnosis of diabetes as recorded by the patient’s healthcare provider (“doctor told you have diabetes”), (2) glycohemoglobin A1c (HbA1c) level of greater than 6.5%, (3) a fasting blood glucose level of equal to or greater than 7.0 mmol/L, (4) a random blood glucose level of equal to or greater than 11.1 mmol/L, or (5) a two-hour blood glucose level of equal to or greater than 11.1 mmol/L following an oral glucose tolerance test (OGTT). Hyperlipidemia was defined by TG levels equal to or greater than 150 mg/dL, hypercholesterolemia, or medication that lowers lipid levels. Individuals who met any of the following criteria were considered to have hypercholesterolemia: (1) TC levels equal to or greater than 200 mg/dL, (2) LDL-C levels equal to or greater than 130 mg/dL, or (3) HDL-C levels less than 40 mg/dL for males and less than 50 mg/dL for females.

### 2.5. Statistical analysis

We initiated our analysis by comparing the baseline data. Based on sex, participants were divided into two groups according to the presence of hyperuricemia for baseline characteristic analysis. Given that different components of the DII might exhibit varying impacts on inflammation and could demonstrate exponential growth or decline, we presented the distribution of different DII components among different groups in the form of medians and interquartile ranges. Mean ± standard deviation was used to report continuous variables, while percentages were used to represent categorical variables. For variables with a normal distribution, analysis was conducted using Student’s t-test or chi-squared test. When variables exhibited skewed distribution, non-parametric tests or Fisher’s exact probability test were employed for analysis. Furthermore, a multivariable logistic regression model was utilized in both the overall population and sex-stratified subgroups to estimate odds ratios and 95% confidence intervals. The DII score was included in the model as an independent variable, both in continuous and tertile forms (T1 (−5.28 to 0.79, *n* = 10,594), T2 (0.79 to 2.60, *n* = 10,593), T3 (2.60 to 5.79, *n* = 10,594)), to explore potential correlations with hyperuricemia. The transformation of DII into tertile variables aims to assess whether DII exhibits correlations with hyperuricemia across different variable type states. Additionally, linear trend tests were conducted to assess linear relationships, and a Generalized Additive Model (GAM) along with smoothed curve fitting and a two-part logistic regression model were employed to explore nonlinear correlations. Subgroup and interaction analyses were performed for covariates such as sex, age, race/ethnicity, hypertension, diabetes, and BMI, while controlling for potential confounding factors. All statistical analyses were conducted using R (version 3.5.3) and EmpowerStats,^1^ with statistical significance defined as *p* < 0.05.

## 3. Results

### 3.1. Baseline characteristics

Table 1 displays the baseline characteristics based on sex for the hyperuricemia and non-hyperuricemia groups. The male group comprises 15,828 individuals, while the female group consists of 15,953 individuals. Among males, age (*p* = 0.067), METs/week (*p* = 0.355), and diabetes (*p* = 0.837) showed no statistically significant differences between the hyperuricemia and non-hyperuricemia groups. However, there were statistically significant differences between the groups in terms of race/ethnicity, education level, smoking, alcohol consumption, BMI, hypertension, hyperlipidemia, and eGFR. Among males, those with lower education levels, higher BMI, higher blood pressure, lower eGFR, history of smoking, moderate-to-heavy alcohol consumption, and higher lipid levels were more prone to hyperuricemia. Furthermore, among the female population, statistically significant differences were observed among all variables.

**Table 1:** Baseline characteristics of subjects.

Characteristics	Male				Female			
	Total *n*=15828	Non-Hyperuricemia *n*=12548	Hyperuricemia *n*=3280	*p*-value	Total *n*=15953	Non-Hyperuricemia *n*=13742	Hyperuricemia *n*=2211	*p*-value
Age (years)	45.9 ± 17.9	46.1 ± 18.1	45.4 ± 17.3	0.067	46.0 ± 17.5	45.0 ± 17.5	52.0 ± 16.8	<0.001
Race/ethnicity (%)				<0.001				<0.001
Non-Hispanic White	41.94	41.73	42.74		39.83	39.69	40.71	
Non-Hispanic Black	20.92	20.29	23.32		21.38	20.16	28.95	
Mexican American	17.10	18.09	13.32		17.18	18.00	12.03	
Others	20.04	19.89	20.61		21.61	22.14	18.32	
Education level (%)				<0.001				0.043
Less than high school	26.08	26.74	23.55		23.49	23.69	22.31	
High school	22.70	22.63	22.97		20.32	20.01	22.22	
More than high school	51.22	50.63	53.48		56.19	56.30	55.48	
BMI (kg/m^2^)	28.55 ± 6.17	27.79 ± 5.65	31.47 ± 7.14	<0.001	29.40 ± 7.65	28.56 ± 7.12	34.65 ± 8.71	<0.001
SBP (mmHg)	124.21 ± 16.15	123.78 ± 16.03	125.87 ± 16.52	<0.001	120.54 ± 18.73	119.58 ± 18.47	126.49 ± 19.24	<0.001
DBP (mmHg)	71.58 ± 12.13	71.01 ± 11.95	73.79 ± 12.60	<0.001	69.28 ± 11.29	69.07 ± 11.11	70.61 ± 12.28	<0.001
Smoking, *n* (%)				<0.001				<0.001
Never	47.34	46.82	49.29		65.07	66.00	59.50	
Former	27.65	27.21	29.33		17.19	16.28	22.66	
Now	25.01	25.96	21.39		17.74	17.72	17.84	
Drinking (%)				<0.001				<0.001
Never	8.44	8.60	7.81		20.18	20.23	19.84	
Former	15.18	15.66	13.33		14.28	13.72	17.60	
Mild	38.06	38.66	35.78		27.98	28.25	26.32	
Moderate	12.42	12.11	13.57		19.89	20.06	18.84	
Heavy	25.91	24.96	29.51		17.69	17.73	17.40	
METs/week (%)				0.355				0.015
Low	19.97	20.06	19.61		27.51	27.14	29.99	
Moderate	11.84	11.63	12.63		16.40	16.26	17.32	
Vigorous	68.20	68.31	67.77		56.09	56.60	52.69	
eGFR (ml/min/1.73 m^2^)	98.23 ± 19.26	99.18 ± 19.12	94.58 ± 19.33	<0.001	101.22 ± 20.45	102.82 ± 20.08	91.27 ± 19.93	<0.001
UA (mg/dl)	5.99 ± 1.26	5.52 ± 0.88	7.79 ± 0.77	<0.001	4.72 ± 1.16	4.39 ± 0.83	6.77 ± 0.77	<0.001
Serum glucose (mg/dl)	102.94 ± 38.40	103.17 ± 40.35	102.10 ± 29.79	<0.001	99.11 ± 35.64	97.99 ± 35.69	106.09 ± 34.50	<0.001
Diabetes (%)	16.53	16.50	16.65	0.837	15.14	12.95	28.81	<0.001
Hypertension (%)	37.98	35.47	47.59	<0.001	36.28	32.31	60.92	<0.001
Hyperlipidemia (%)	66.96	64.70	75.61	<0.001	70.11	67.75	84.80	<0.001
DII	1.15 ± 1.88	1.10 ± 1.89	1.35 ± 1.84	<0.001	1.87 ± 1.80	1.84 ± 1.81	2.08 ± 1.73	<0.001

BMI, body mass index; SBP, systolic blood pressure; DBP, diastolic blood pressure; MET, metabolic equivalent of task; eGFR, estimated glomerular filtration rate; UA, uric acid; DII, dietary inflammatory index.

Additionally, Table 2 presents the scores for each component of the DII based on sex-specific grouping for the hyperuricemia and non-hyperuricemia groups. From Table 2, it can be observed that, for both males and females, there are no statistically significant differences in cholesterol intake and caffeine intake between the groups. Among males, there are no statistically significant differences in vitamin B6 and niacin intake between the hyperuricemia and non-hyperuricemia groups. Among females, there are no statistically significant differences in β-Carotene intake between the groups. However, for the other components of the DII, statistically significant differences are observed between the hyperuricemia and non-hyperuricemia groups for both males and females.

**Table 2:** Comparison of each component of DII scores between individuals with hyperuricemia and individuals without hyperuricemia in different sexes.

DII components	Male				Female			
	Total *n* = 15,828	Non-Hyperuricemia *n* = 12,548	Hyperuricemia *n* = 3,280	*p*-value	Total *n* = 15,953	Non-Hyperuricemia *n* = 13,742	Hyperuricemia *n* = 2,211	*p*-value
Energy	0.103 (−0.120–0.179)	0.108 (−0.117–0.180)	0.078 (−0.130–0.179)	<0.001	−0.125 (−0.176–0.066)	−0.122 (−0.175–0.069)	−0.144 (−0.177–0.036)	<0.001
Protein	0.010 (−0.016–0.021)	0.010 (−0.015–0.021)	0.008 (−0.017–0.021)	0.002	−0.016 (−0.021–0.007)	−0.015 (−0.021–0.008)	−0.016 (−0.021–0.005)	0.034
Carbohydrate	−0.001 (−0.091–0.095)	0.006 (−0.089–0.096)	−0.027 (−0.094–0.091)	<0.001	−0.085 (−0.097–0.009)	−0.084 (−0.097–0.014)	−0.092 (−0.097--0.026)	<0.001
Dietary fiber	0.287 (−0.453–0.608)	0.249 (−0.484–0.595)	0.425 (−0.296–0.633)	<0.001	0.490 (−0.054–0.636)	0.478 (−0.075–0.634)	0.553 (0.118–0.646)	<0.001
Total fatty acid	0.154 (−0.153–0.294)	0.165 (−0.146–0.295)	0.121 (−0.180–0.290)	<0.001	−0.094 (−0.256–0.190)	−0.091 (−0.255–0.194)	−0.113 (−0.265–0.164)	0.001
Total saturated fatty acid	−0.057 (−0.314–0.312)	−0.042 (−0.309–0.321)	−0.126 (−0.331–0.269)	<0.001	−0.271 (−0.354–0.016)	−0.268 (−0.354–0.025)	−0.290 (−0.358--0.041)	<0.001
MUFA	−0.004 (−0.009–0.007)	−0.004 (−0.009–0.006)	−0.003 (−0.009–0.007)	<0.001	0.005 (−0.005–0.009)	0.005 (−0.005–0.009)	0.005 (−0.004–0.009)	0.003
PUFA	−0.247 (−0.337–0.153)	−0.252 (−0.337–0.146)	−0.228 (−0.337–0.184)	0.012	−0.016 (−0.318–0.276)	−0.022 (−0.319–0.273)	0.026 (−0.315–0.288)	0.008
Cholesterol	−0.006 (−0.108–0.110)	−0.002 (−0.107–0.110)	−0.014 (−0.108–0.110)	0.096	−0.101 (−0.110–0.070)	−0.101 (−0.110–0.070)	−0.101 (−0.110–0.070)	0.472
Vitamin A	0.264 (0.098–0.335)	0.257 (0.084–0.332)	0.290 (0.154–0.343)	<0.001	0.285 (0.156–0.341)	0.283 (0.153–0.340)	0.296 (0.181–0.347)	<0.001
Vitamin B1	0.006 (−0.062–0.058)	0.002 (−0.065–0.056)	0.020 (−0.046–0.065)	<0.001	0.050 (−0.000–0.078)	0.049 (−0.002–0.077)	0.058 (0.011–0.081)	<0.001
Vitamin B2	−0.026 (−0.060–0.017)	−0.028 (−0.061–0.015)	−0.016 (−0.054–0.025)	<0.001	0.007 (−0.034–0.037)	0.005 (−0.035–0.036)	0.015 (−0.023–0.041)	<0.001
Vitamin B6	−0.205 (−0.348–0.024)	−0.206 (−0.349–0.023)	−0.200 (−0.345–0.034)	0.239	−0.004 (−0.226–0.175)	−0.008 (−0.228–0.172)	0.016 (−0.204–0.191)	<0.001
Vitamin B12	−0.020 (−0.070–0.064)	−0.016 (−0.069–0.067)	−0.029 (−0.074–0.051)	<0.001	−0.057 (−0.084–0.001)	--0.056 (−0.084–0.003)	−0.062 (−0.086--0.006)	<0.001
Vitamin C	0.362 (−0.065–0.413)	0.357 (−0.087–0.413)	0.379 (0.024–0.415)	<0.001	0.374 (0.076–0.414)	0.370 (0.057–0.413)	0.390 (0.180–0.415)	<0.001
Vitamin D	00.359 (−0.055–0.435)	0.352 (−0.086–0.434)	0.394 (0.070–0.438)	<0.001	0.394 (0.150–0.438)	0.394 (0.135–0.437)	0.410 (0.232–0.438)	<0.001
Vitamin E	0.271 (−0.375–0.416)	0.255 (−0.385–0.416)	0.317 (−0.322–0.418)	<0.001	0.389 (−0.089–0.419)	0.387 (−0.101–0.418)	0.402 (0.012–0.419)	<0.001
β-Carotene	0.537 (0.388–0.557)	0.537 (0.380–0.557)	0.540 (0.414–0.559)	0.004	0.536 (0.340–0.558)	0.536 (0.342–0.558)	0.537 (0.322–0.558)	0.697
Niacin	−0.016 (−0.164–0.110)	−0.016 (−0.165–0.110)	−0.016 (−0.161–0.110)	0.749	0.110 (−0.000–0.177)	0.109 (−0.001–0.176)	0.117 (0.004–0.181)	0.016
Folate	0.172 (0.011–0.189)	0.169 (−0.009–0.189)	0.180 (0.074–0.189)	<0.001	0.185 (0.130–0.190)	0.185 (0.125–0.190)	0.187 (0.151–0.190)	<0.001
Magnesium	0.014 (−0.253–0.231)	0.006 (−0.261–0.224)	0.050 (−0.224–0.259)	<0.001	0.184 (−0.036–0.321)	0.179 (−0.041–0.318)	0.222 (0.014–0.340)	<0.001
Iron	0.011 (−0.018–0.031)	0.013 (−0.017–0.031)	0.003 (−0.022–0.029)	<0.001	−0.013 (−0.027–0.015)	−0.013 (−0.027–0.017)	−0.018 (−0.028–0.008)	<0.001
Zinc	−0.184 (−0.312–0.190)	−0.199 (−0.312–0.181)	−0.114 (−0.311–0.223)	<0.001	0.155 (−0.192–0.292)	0.148 (−0.200–0.291)	0.193 (−0.134–0.299)	<0.001
Selenium	−0.184 (−0.191--0.098)	−0.185 (−0.191--0.101)	−0.182 (−0.191--0.089)	0.012	−0.110 (−0.184–0.035)	−0.112 (−0.184–0.032)	−0.096 (−0.181–0.044)	0.003
Caffeine	0.084 (0.083–0.085)	0.084 (0.083–0.085)	0.084 (0.083–0.085)	0.508	0.084 (0.084–0.085)	0.084 (0.084–0.085)	0.084 (0.084–0.085)	0.574
Alcohol	0.278 (−0.001–0.278)	0.278 (0.136–0.278)	0.278 (−0.278–0.278)	<0.001	0.278 (0.278–0.278)	0.278 (0.278–0.278)	0.278 (0.278–0.278)	<0.001
n3 Polyunsaturated fatty acid	0.287 (0.273–0.294)	0.287 (0.273–0.294)	0.286 (0.271–0.294)	0.003	0.290 (0.279–0.295)	0.290 (0.279–0.295)	0.290 (0.278–0.295)	0.024
n6 Polyunsaturated fatty acid	−0.102 (−0.153--0.010)	−0.103 (−0.153--0.012)	−0.096 (−0.152--0.000)	0.003	−0.050 (−0.130–0.036)	−0.052 (−0.130–0.035)	−0.041 (−0.127–0.044)	0.002

Data are presented as the median and interquartile ranges (Q1-Q3). DII, dietary inflammatory index; MUFA, monounsaturated fatty acids; PUFA, polyunsaturated fatty acids.

### 3.2. Association between DII score and hyperuicemia

**Figure 2:**
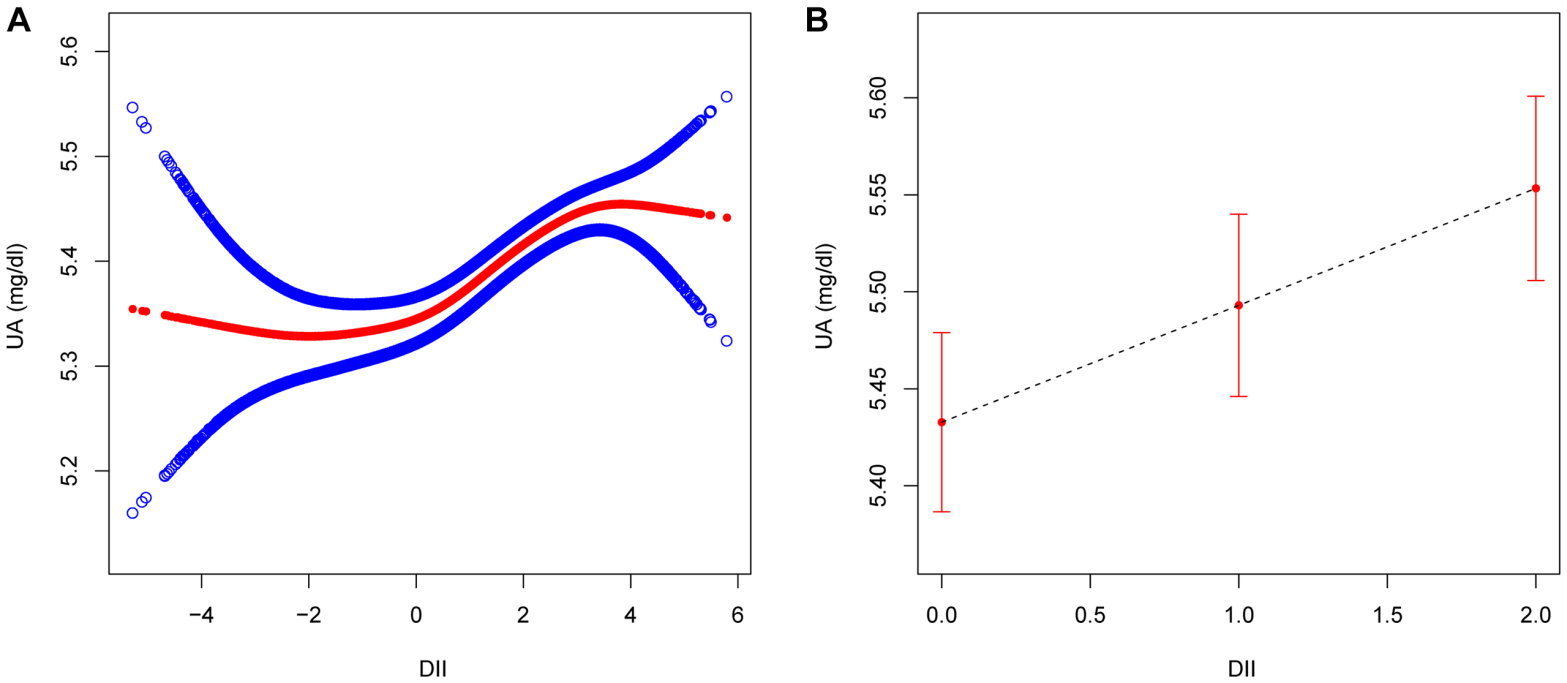
The association between DII (**(A)** as continuous variable; **(B)** as categorical variable) and UA. Age, sex, BMI, race/ethnicity, educational level, smoking, drinking, MET, eGFR, diabetes, hypertension, and hyperlipidemia were adjusted. DII, dietary inflammatory index; UA, uric acid; BMI, body mass index; MET, metabolic equivalent of task; eGFR, estimated glomerular filtration rate.

The logistic regression modeling results shown in Table 3 indicates the association between DII score and hyperuricemia. After adjusting for all covariates (age, sex, BMI, race/ethnicity, educational level, smoking, drinking, MET, eGFR, diabetes, hypertension, and hyperlipidemia), a positive association was established between these two (OR 1.06, 95% CI 1.04–1.09). The second and third tertile groups showed higher odds than the first tertile (T2: OR 1.17, 95% CI 1.07–1.29; T3: OR 1.31, 95% CI 1.19–1.44), which was also confirmed by the trend test (*p*<0.05). At the same time, we also conducted sex based stratified analysis and found that the relationship between DII and hyperuricemia remained unchanged among different sexes. Additionally, we used a GAM and a smooth curve fit to evaluate the correlation between them and evaluated their nonlinearity using a two part logistic regression model. When DII was treated as a continuous variable, a positive correlation was observed between DII and uric acid (Figure 2A). When DII was treated as a categorical variable with three tertiles, the relationship between DII and uric acid remained unchanged (Figure 2B). The results of a two part logistic regression model also show that there is no nonlinear relationship between DII and uric acid (Supplementary Table S1).

**Table 3:** Odd ratios and 95% confidence intervals for hyperuricemia according to DII.

Characteristics	Model 1	Model 2	Model 3
Total (*n* = 31,781)			
Continuous	1.05 (1.03, 1.06)	1.05 (1.04, 1.07)	1.06 (1.04, 1.09)
DII Tertile			
T1 (−5.28, 0.79)	Reference	Reference	Reference
T2 (0.79, 2.60)	1.15 (1.07, 1.23)	1.14 (1.06, 1.23)	1.17 (1.07, 1.29)
T3 (2.60, 5.79)	1.21 (1.12, 1.30)	1.26 (1.17, 1.36)	1.31 (1.19, 1.44)
P for trend	<0.001	<0.001	<0.001
Male (*n* = 15,828)			
Continuous	1.07 (1.05, 1.10)	1.06 (1.04, 1.08)	1.07 (1.05, 1.10)
DII Tertile			
T1 (−5.28, 0.79)	Reference	Reference	Reference
T2 (0.79, 2.60)	1.24 (1.13, 1.36)	1.18 (1.07, 1.29)	1.27 (1.13, 1.42)
T3 (2.60, 5.79)	1.34 (1.21, 1.47)	1.27 (1.15, 1.40)	1.37 (1.21, 1.56)
P for trend	<0.001	<0.001	0.007
Female (*n* = 15,953)			
Continuous	1.08 (1.05, 1.11)	1.06 (1.03, 1.09)	1.05 (1.02, 1.09)
DII Tertile			
T1 (−5.28, 0.79)	Reference	Reference	Reference
T2 (0.79, 2.60)	1.16 (1.03, 1.32)	1.11 (0.97, 1.26)	1.03 (0.88, 1.22)
T3 (2.60, 5.79)	1.38 (1.23, 1.55)	1.28 (1.13, 1.44)	1.23 (1.05, 1.44)
P for trend	<0.001	<0.001	<0.001

Model 1: Non-adjusted. Model 2: Adjusted for age, sex, and BMI. Model 3: Adjusted for age, sex, BMI, race/ethnicity, educational level, smoking, drinking, MET, eGFR, diabetes, hypertension, and hyperlipidemia. The sex variables were not adjusted in the stratified analysis of sex. DII, dietary inflammatory index; BMI, body mass index; MET, metabolic equivalent of task; eGFR, estimated glomerular filtration rate.

**Figure 3:**
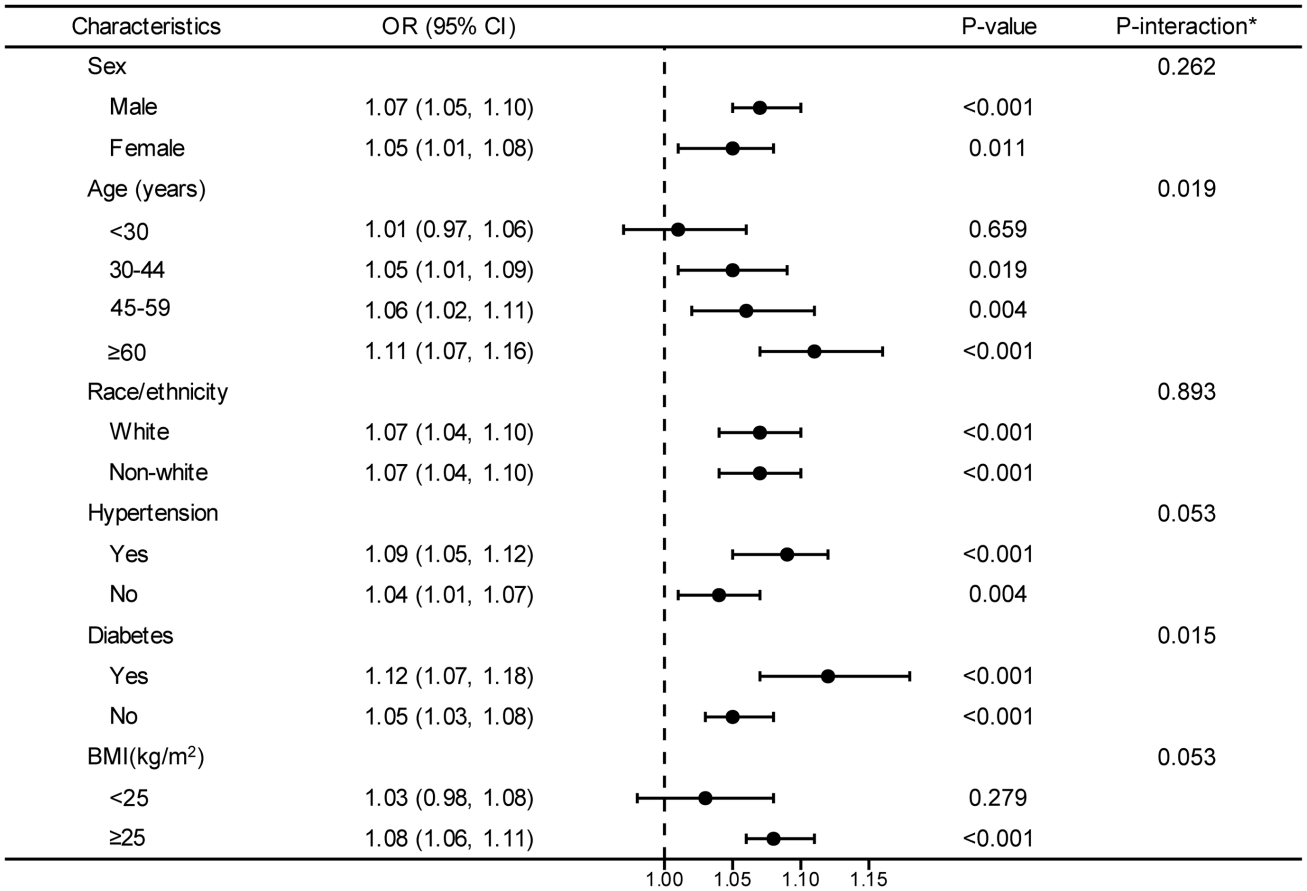
Stratified analyses between DII and hyperuricemia. OR values are based on different population stratifications, estimated using a multivariable logistic regression model to assess the odds ratios and 95% confidence intervals between DII and hyperuricemia. The first column of *p*-values represents the significance of OR values after stratification by different population groups. The second column of P-values pertains to the significance of models considering interaction terms between DII and various variables (sex, age, race/ethnicity, hypertension, diabetes and BMI). ^*^Each stratification adjusted for all the factors (age, sex, BMI, race/ethnicity, educational level, smoking, drinking, MET, eGFR, diabetes, hypertension, and hyperlipidemia) except the stratification factor itself. OR, odd ratio; CI, confidence interval; DII, dietary inflammatory index; BMI, body mass index; MET, metabolic equivalent of task; eGFR, estimated glomerular filtration rate.

The forest plot illustrates interactions between DII and hyperuricemia concerning age and diabetes (*p* < 0.05). Borderline significance levels for interactions in hypertension and BMI are observed at a *p* value of 0.05. However, interactions between DII and hyperuricemia are not significant for sex and race/ethnicity (*p* > 0.05) (Figure 3).

## 4. Discussion

In this cross-sectional study using NHANES data, we found that DII, which is an indicative index for pro-inflammatory diet, was in significant positive association with hyperuricemia after adjusting for multiple covariates. Compared with the lowest tertile subgroup of the DII, the highest tertile subgroup of the DII increased the risk of hyperuricemia by 31% for all participants, including 37 and 23% for male and female, respectively. Likewise, this positive correlation has also been validated in the GAM and through smooth curve fitting.

The results between the DII components and hyperuricemia showed that both male and female, the hyperuricemia group had higher intakes of dietary fiber, PUFA, vitamin A, vitamin B1, vitamin B2, vitamin B12, vitamin C, vitamin D, vitamin E, folate, magnesium, zinc, selenium, and n6 polyunsaturated fatty acids, as compared with the non-hyperuricemia group. In addition, female participants had higher levels of vitamin B6 and niacin intake in the hyperuricemia group than in the non-hyperuricemia group; and the male hyperuricemia group had higher levels of beta-carotene intake than the non-hyperuricemia group. Moreover, the differences between the cholesterol and caffein were not significant in either male or female. Hyperuricemia group had a slightly higher intake of dietary fiber than the other. The possible mechanism behind might be that the viscosity and bulk of dietary fiber interfere with the absorption of purines or adenine in the digestive system (17), or enhance intestinal motility and potentially binding uric acid in the intestine to facilitate its excretion (18). Additionally, In addition, our study found significant differences in certain vitamins in DII between the hyperuricemia and non-hyperuricemia groups, regardless of sex differences, which these vitamins are associated with hyperuricemia in various ways. For example, vitamin C mediates serum uric acid level by effectively preventing the impairment of renal epithelial function caused by uric acid crystals (19). Vitamin B12, as summarized by Zhang et al., its combination with folic acid could inactivate xanthine oxidoreductase, interfere with the conversion of purines to uric acid, and also reduce homocysteinemia level that may induce significant DNA damage and release purine nucleotides, ultimately reducing uric acid (20). Vitamin D affects the secretion and transport of uric acid by influencing serum parathyroid hormone level. Vitamin E acts by inhibiting xanthine oxidase activity and reducing uric acid formation (21). In addition, an experimental study reported that vitamin E also promotes uric acid excretion in deoxycorticosterone-salt-treated rats (22). In the end, a significant difference was also identified in alcohol intake between two groups. In addition, in this study we observed that cholesterol and caffeine intake did not differ significantly between the hyperuricemia and non-hyperuricemia groups of male and female, which may be related to the prevalence of caffeine consumption as well as to the Western dietary pattern in the US, which is characterized by a high-calorie and fat diet and is usually associated with higher levels of inflammatory markers in the body (9, 23, 24). Most Americans follow this pattern of dietary consumption, eliminating or diminishing its role between the sexes.

In addition, most people tend to consume food in several dietary patterns or their combinations. Current assessment only focused on the effect of single nutrient, which may lead to one-sided outcomes due to its neglect on synergistic effects of nutritional patterns (25). Further regression analysis showed that there is a positive association between DII and hyperuricemia risk after adjusting for potential confounders, with similar associations for male and female participants. A study among a Chinese population indicated a highly positive correlation between DII score and serum uric acid levels, regardless of sex (14). While, a Korean study found that only female with higher DII scores had a higher risk of HUA (26). The discrepancy could be partially due to the differences in the eating habits of different populations. The Korean diet is characterized by containing large amounts of fermented foods and seafood (27). In contrast to the dietary habits in Korea, Western diets are characterized by high fat intake, which contain processed meats and red meats (24). According to the DII concept (12), a pro-inflammatory diet contains mainly of red and processed meats, fried foods, high-sugar foods and refined grains, which are associated with high uric acid levels (28). An anti-inflammatory diet includes more vegetables, fruits, soy products, whole grains and nuts. Therefore, a healthy diet might be able to reduce the risk of hyperuricemia.

Previous studies have confirmed that serum uric acid level is determined by the production-excretion balance of uric acid, in which excretion via kidney plays a major role (29). In addition, it has been shown that intestine, other than kidney, is an important potential organ for the excretion of uric acid, and it works primarily through the action of intestinal flora and uric acid transporters (30). Thus the potential mechanism by which DII influence hyperuricemia are as follows. DII is closely associated with the development of hyperuricemia through direct effects of host purine metabolism or indirect effect of gut microbiota. On one hand, diets that promote inflammation can have adverse effects on an individual’s health by inducing oxidative stress and disrupting the immune system. Such diets can significantly increase the levels of inflammatory cytokines, exacerbating the problem. Additionally, they could alter the gastrointestinal microbial ecosystem, leading to chronic inflammation and other related health issues (31). Chronic inflammation is one of the main mechanism causing renal injury, which may ultimately impede uric acid excretion (32). On the other hand, anti-inflammatory diets in DII downregulate adenosine deaminase and xanthine oxidase activities, and improve intestinal barrier function and restores intestinal microbiota metabolism (33). Therefore, it might be a new therapeutic strategy targeting at metabolic balance of purines along with the regulation of intestinal excretion of uric acid, to prevent and alleviate hyperuricemia in the future.

In the final forest plot, our study indicates that there is an interaction between age and diabetes concerning the relationship between DII and hyperuricemia (p value for interaction <0.05). Additionally, blood pressure and BMI might also have a certain influence on the association between DII and hyperuricemia (borderline significant p value for interaction). Previous study pointed out that the population of participants who were ≥ 60 years old had the highest hyperuricemia risk (34). Possible explanations for this discrepancy could be attributed to different leptins levels in different age groups. It has been shown that leptin plays a critical role in inflammatory and immune responses (35), and is an important independent variable of uric acid values across all age groups (36). Also, a study by Francesco et al. showed that fasting leptin was higher in elderly subjects than in younger subjects, even after adjusting for the covariate of fat mass (37). Based on these studies, it is reasonable to deduce that the age-based DII-hyperuricemia relationship might be influenced by leptin levels. Additionally, we found that DII and hyperuricemia had a stronger association in patients with diabetes, which was quite expectable and was consistent with other studies which have confirmed a linear and positive correlation between serum uric acid levels in diabetes mellitus and serum insulin levels (38). A longitudinal study had reported that patients with diabetes at baseline were related to an increased hyperuricemia risk (39). Diabetes may regulate the association between DII and hyperuricemia by insulin resistance. Insulin resistance is a prevalent medical condition that could potentially cause an upsurge in the activity of hexose monophosphate shunt. This could lead to an augment in purine biosynthesis and turnover, ultimately resulting in an increase in the level of uric acid (40). Moreover, we found that as DII scores increase, the risk of hyperuricemia in individuals with a BMI > 25 also increases. This is consistent with a previous analysis of a representative sample of U.S. adults, which indicated that obesity plays a mediating role in the relationship between diet and hyperuricemia, with an indirect effect proportion of BMI as high as 53.34% (41). In the meanwhile, some studies have reached conclusions that are inconsistent with ours. A Korean case–control study evaluating a pro-inflammatory diet and the risk of hyperuricemia found that participants with a BMI <25 had a higher risk of developing hyperuricemia (26, 42). Additionally, research suggests that not only overweight but also being underweight is associated with higher levels of inflammation. This could explain why low BMI is also correlated with an increased risk of hyperuricemia (43, 44). It is worth emphasizing that these studies are currently based on studies conducted in single-country populations, leading to limitations in the extrapolation of findings. Meanwhile, similar to our findings, a survey from the U.S. indicated participants with hypertension had an elevated DII compared with those without hypertension (45). Although the underlying mechanisms are not clear, it is possible that the underlying hypertension risk or improved dietary pattern of these participants played a partial role in the correlation between DII and HUA risk (46). Hence, further investigation is needed to elucidate the question of whether hypertension might play a role in the relationship between DII and hyperuricemia. In contrast, a study showed a positive association between DII score and hyperuricemia that did not vary by hypertension status. The discrepancy could be partially due to the criteria for inclusion and exclusion, and sample size of the study participants. In our study, to minimize the potential adverse effects of renal impairment on nutritional intake, we only included individuals with an eGFR of ≥60 mL/min/1.73 m (2). In terms of sample size, we incorporated a larger number of samples (n = 31,781 VS n = 19,004), which might provide a more representative reflection of the characteristics of a larger population.

There are some advantages and limitations of this study. This is the first study to confirm the association between DII and hyperuricemia using data from a large-scale U.S. adults based on NHANES. However, present results should be interpreted with caution as cross-sectional observational studies cannot demonstrate causation and directionality. Secondly, although confounding factors have been extensively adjusted, other elements cannot be completely ruled out. Besides this, a possible limitation is the self-reporting of some conditions by the study participants. Information about self-reported may be recall biased or interview subjects diagnosed with hyperuricemia might change their diet pattern. Hyperuricemia represents a major global public health burden, thus further longitudinal studies should be conducted to provide stronger evidence for the relationship between DII and hyperuricemia.

## 5. Conclusion

In conclusion, our study based on an existing database highlighted the association between a diet with higher inflammatory potential measured by DII and increased risk of developing hyperuricemia. These findings suggest that adjusting dietary habits to reduce inflammation may be an effective prevention and control strategy for hyperuricemia. This study provides valuable evidence for healthcare professionals and individuals to consider when making dietary choices to maintain optimal health. Future research aiming to perform interventions based on dietary modifications may yield promising results for combating hyperuricemia and other related health conditions.

## Data availability statement

The raw data supporting the conclusions of this article will be made available by the authors, without undue reservation.

## Ethics statement

All data came from NHANES, which was approved by National Centre for Health Statistics Institutional Ethics Review Board, and all the subjects agreed on the survey and signed written consent. The studies were conducted in accordance with the local legislation and institutional requirements. The participants provided their written informed consent to participate in this study.

## Author contributions

HW: writing-most of manuscript, data curation and processing. SQ: writing-part of the manuscript and data curation. FL: writing-part of the manuscript. HZ: software, writing—review and editing. LZ: methodology, writing—review and editing, and supervision. All authors contributed to the article and approved the submitted version.

## Conflict of interest

The authors declare that the research was conducted in the absence of any commercial or financial relationships that could be construed as a potential conflict of interest.

## Publisher’s note

All claims expressed in this article are solely those of the authors and do not necessarily represent those of their affiliated organizations, or those of the publisher, the editors and the reviewers. Any product that may be evaluated in this article, or claim that may be made by its manufacturer, is not guaranteed or endorsed by the publisher.

## Footnotes

^1^ http://www.EmpowerStats.com
