# Supplementary material for: A cross-sectional study on the association between dietary inflammatory index and hyperuricemia based on NHANES 2005–2018
Source: Front Nutr. 2023 Sep 21;10:1218166. doi: 10.3389/fnut.2023.1218166 (PMC10552180; doi:10.3389/fnut.2023.1218166)
Supplement: Supplementary file 1 [file Data_Sheet_1.docx]

**Table S1. Threshold effect analysis of DII on UA using a two-part logistic regression model**

| **DII** | **Adjusted OR^*^ (95% CI)** | **P-value** |
| --- | --- | --- |
| **Model I** |  |  |
| Fitting by the standard linear model | 0.02 (0.02, 0.03) | <0.05 |
| **Model II** |  |  |
| Inflection point | 3.66 |  |
| < 3.66 | 0.03 (0.02, 0.04) | <0.05 |
| > 3.66 | -0.06 (-0.15, 0.03) | 0.17 |
| **Log likelihood ratio** | / | 0.06 |

**Notes:** ^*^Adjusted for age, sex, BMI, race/ethnicity, educational level, smoking, drinking, MET, eGFR, diabetes, hypertension, and hyperlipidemia.

**Abbreviations:** DII, dietary inflammatory index; UA, uric acid; BMI, body mass index; MET, metabolic equivalent of task; eGFR, estimated glomerular filtration rate.
